# Long-term Immunogenicity and Boostability of the 13-valent Pneumococcal Conjugate Vaccine Followed by the 23-valent Pneumococcal Polysaccharide Vaccine in Adults Receiving Immunosuppressive Therapy and Adults With HIV—3-year Follow-up of a Prospective Cohort Study

**DOI:** 10.1093/cid/ciaf438

**Published:** 2025-08-06

**Authors:** Jenny L Schnyder, Sabine Haggenburg, Hannah M Garcia Garrido, Anne Reiners, Bob Meek, Martin P Grobusch, Abraham Goorhuis

**Affiliations:** Centre for Tropical Medicine and Travel Medicine, Department of Infectious Diseases, Division of Internal Medicine, Amsterdam UMC, Location University of Amsterdam, Amsterdam, The Netherlands; Centre for Tropical Medicine and Travel Medicine, Department of Infectious Diseases, Division of Internal Medicine, Amsterdam UMC, Location University of Amsterdam, Amsterdam, The Netherlands; Department of Experimental Immunology, Department of Haematology, Amsterdam Institute for Infection and Immunity, Amsterdam UMC, Location University of Amsterdam, Amsterdam, The Netherlands; Centre for Tropical Medicine and Travel Medicine, Department of Infectious Diseases, Division of Internal Medicine, Amsterdam UMC, Location University of Amsterdam, Amsterdam, The Netherlands; Department of Medical Microbiology and Immunology, St. Antonius Hospital, Nieuwegein, The Netherlands; Department of Medical Microbiology and Immunology, St. Antonius Hospital, Nieuwegein, The Netherlands; Centre for Tropical Medicine and Travel Medicine, Department of Infectious Diseases, Division of Internal Medicine, Amsterdam UMC, Location University of Amsterdam, Amsterdam, The Netherlands; Centre for Tropical Medicine and Travel Medicine, Department of Infectious Diseases, Division of Internal Medicine, Amsterdam UMC, Location University of Amsterdam, Amsterdam, The Netherlands

**Keywords:** pneumococcal vaccines, immunocompromised host, autoimmune diseases, HIV, immunogenicity

## Abstract

**Background:**

The long-term immunogenicity of the 13-valent pneumococcal conjugate vaccine (PCV13) followed by the 23-valent pneumococcal polysaccharide vaccine (PPSV23) remains unclear among immunocompromised patients (ICPs).

**Methods:**

This 3-year follow-up of a previously reported prospective cohort study included people with human immunodeficiency virus (HIV) (PWH), patients on immunosuppressive therapy, and immunocompetent controls who received PCV13 followed by PPSV23 2 months later. IgG levels for all 24 vaccine serotypes were measured 3 years after PCV13 (M36). The primary outcome was the seroprotection rate (SPR) at M36, defined as IgG concentrations ≥1.3 μg/mL for 17/24 vaccine serotypes. To assess immunological memory, we measured rapid recall responses 7 days after a PCV20 booster vaccination among initial responders 2 months after the priming schedule (M4) who had sero-reverted at M36.

**Results:**

Between M4 to M36, SPRs dropped from 44% (22/50) to 9% (5/55) in PWH, from 55% (59/108) to 17% (22/131) in patients on immunosuppressive therapy and from 82% (14/17) to 42% (8/19) in controls. Rapid recall responses were observed in 40% (4/10) of PWH, 14% (2/14) of patients on immunosuppressive monotherapy, 22% (2/9) of patients on combination therapy, and 67% (2/3) of controls. Antibody levels increased significantly for 7/13 PCV20/PCV13-shared serotypes, but for none (0/7) of the PCV20/PPSV23-shared serotypes.

**Conclusions:**

Only a minority of PWH and patients on immunosuppressive therapy and under half of controls remained seroprotected 3 years after vaccination. As rapid recall responses were limited to PCV serotypes, future research in ICPs should focus on expanded priming schedule with higher-valent PCVs such as PCV20.

## Teaser

Three years post-PCV13/PPSV23 the majority of PWH and patients on immunosuppressive therapy, and over half of controls lost seroprotection. Rapid recall responses were limited to PCV serotypes, supporting the need for improved priming schedules with higher-valent conjugate vaccines.

*Streptococcus pneumoniae* is a major global cause of morbidity and mortality, responsible for over 1 million deaths annually [1]. Immunocompromised patients (ICPs) are at increased risk of both invasive pneumococcal disease (IPD) and community-acquired pneumonia (CAP) with *S. pneumoniae* [2–5]. In patients with autoimmune diseases and in solid organ transplant recipients, for whom immunosuppressive therapy is the cornerstone of treatment, a 6.5- and 47-times higher risk of IPD has been reported, respectively [5]. Similarly, people with human immunodeficiency virus (HIV) (PWH), have a >7 times higher risk of IPD and a >8 times higher risk of CAP [2].

Up to 2021, international guidelines recommended pneumococcal vaccination with the 13-valent pneumococcal conjugate vaccine (PCV13; Prevenar 13®), followed 2 months later by the 23-valent polysaccharide vaccine (PPSV23; Pneumovax 23®) for ICPs, including PWH and patients on immunosuppressive therapy [6–8]. In 2021, a 20-valent pneumococcal conjugate vaccine (PCV20; Prevenar 20®) was licensed in the United States, which became available in the Netherlands in 2023 [9]. Since 2023, the CDC has recommended PCV20 for ICPs who received PCV13 and PPSV23 more than 5 years prior [6]. Although the combined PCV13/PPSV23 schedule is immunogenic among ICPs, responses are lower than in controls and immunity wanes rapidly in PWH and patients on immunosuppressive therapy within the first year [10, 11]. However, the long-term durability of humoral immunity after PCV13/PPSV23 has not been studied. Moreover, no studies have assessed PCV20 in ICPs.

This 3-year follow-up of an earlier prospective cohort study (NL7193) [10, 11] investigated serological responses 3 years after priming, to all 24 serotypes included in the PCV13/PPSV23 schedule, in PWH, patients on immunosuppressive therapy and controls. Additionally, we assessed immunological memory via rapid recall responses following a PCV20 booster in initial responders who had sero-reverted 3 years post-PCV13/PPSV23.

## METHODS

### Study Population

As previously reported [10, 11], PWH, patients on immunosuppressive therapy and immunocompetent controls, who visited the Amsterdam UMC vaccination clinic, were included. Patients on immunosuppressive therapy were grouped into: (1) conventional immunomodulators (cIM); (2) biological immunomodulators (bIM); (3) combination therapy; and (4) the “switched group” (initiating therapy between 2 weeks post-PCV13 and 2 weeks post-PPSV23). Participants had received 1 PCV13-dose followed by 1 PPSV23-dose with a 2-month interval. PCV13 includes purified capsular polysaccharide of 13 *S. pneumoniae* serotypes (1, 3, 4, 5, 6A, 6B, 7F, 9V, 14, 18C, 19A, 19F, and 23F) conjugated to CRM197, a nontoxic diphtheria toxin variant. PPSV23 contains purified capsular polysaccharide of 23 serotypes (1, 2, 3, 4, 5, 6B, 7F, 8, 9N, 9V, 10A, 11A, 12F, 14, 15B, 17F, 18C, 19A, 19F, 20, 22F, 23F, and 33F). Antibodies had been measured at months 0, 2, 4, 6, and 12 (M0-M12). All participants from the previous study were eligible for this follow-up study and contacted by e-mail and phoned up to 3 times to ask to participate.

### Study Procedures

Serum samples were collected 3 years post-PCV13 (M36). Serum concentrations of serotype-specific pneumococcal IgG for all 24 combined PCV13/PPSV23 serotypes were measured as previously described [10, 11]. A good serological response was defined as a IgG concentration of ≥1.3 µg/mL for at least 70% (17/24) of PCV13/PPSV23 serotypes [12]. To assess immunological memory, PCV20 was administered to participants who had yielded a good serological response at M4 (peak antibody levels), but lost protection at M36 (sero-reversion). Rapid recall responses were assessed by measuring antibodies at PCV20 administration (DB0) and 7 days later (DB7), as an early rise in antibody levels at this time point is considered a proxy for pre-existing immunological memory (Supplementary Figure 1). A rapid recall response was defined as a good serological response at DB7.

### Study Outcomes

The primary outcome was the overall seroprotection rate (SPR) at M36, defined as the proportion of individuals with a IgG concentration of ≥1.3 µg/mL for at least 70% (17/24) of combined PCV13/PPSV23 serotypes, as recommended by the American Academy of Allergy, Asthma and Immunology (AAAAI) [12]. As consensus on a correlate of protection is lacking for this population, we also displayed our results according to alternative seroprotection cutoffs. A cutoff of ≥1.0 µg/mL, as proposed by Andrews et al [13], was used to define a sufficient serological response. A cutoff of ≥0.35 µg/mL, the World Health Organization (WHO)-recommended correlate in infants [14], was used to define a moderate serological response. A poor serological response was defined as an IgG concentration <0.35 µg/mL. Secondary exploratory outcomes included: (1) the difference in SPR between M4 and M36; (2) SPRs for the group of serotypes in PCV13 (1, 3, 4, 5, 6A, 6B, 7F, 9V, 14, 18C, 19A, 19F, and 23F) and for PPVS23-unique serotypes (2, 8, 9N, 10A, 11A, 12F, 15B, 17F, 20, 22F, and 33F) at M36; (3) serotype-specific median IgG concentrations for all 24 serotypes at M36; (4) median fold antibody concentration changes between M4 and M36 for all 24 serotypes; (5) predictors for the primary outcome; and (6) rapid recall responses following PCV20 booster at DB7, presented for the 3 aforementioned cutoffs of seroprotection.

### Statistical Analysis

No formal sample size calculation was performed as the aim was to include as many former participants as possible. Data were described using means (standard deviations [SD]), medians (interquartile ranges [IQR]) and proportions (%), as appropriate. Groups were compared using ANOVA for normally distributed numerical data, Kruskal–Wallis for non-normally distributed data, χ^2^ for dichotomous data, and McNemar's test for paired dichotomous data. Associations between predictors and the primary outcome among PWH and patients on immunosuppressants were estimated using univariable and multivariable logistic regression. Predictors were selected based on prior literature and stepwise backward selection. Some patients with low antibody levels at M12 had received additional PCV13 and/or PPSV23 booster doses between M12 and M36, based on clinical grounds. Sensitivity analyses were performed to assess whether excluding patients who received a booster dose or those on anti-CD20 (B-cell depleting) therapy, known to impair serological responses more profoundly, would alter the results. Data analyses were conducted using IBM SPSS Statistics version 28, GraphPad Prism version 10.2.0, and R version 4.3.2 [15–17]. Analyses were performed per protocol, including only participants with serum samples collected at both M0 and M36 in the final analysis.

### Ethical Considerations

The study complied with the Declaration of Helsinki and the Medical Research Involving Human Subjects Act (WMO). In 2018, the Amsterdam UMC research ethics committee provided ethical clearance (NL65687.018.18), with amendments for the 3-year follow-up (2021) and PCV20 booster (2024). All participants provided separate written informed consent for the follow-up study.

## RESULTS

Of the initial 298 participants, 205 (69%) individuals were enrolled in this follow-up study, including 55 PWH, 131 patients on immunosuppressive therapy and 19 controls (Supplementary Figure 2). At baseline, PWH were older, more often male, and more likely to use drugs or smoke. Five PWH and 6 patients on immunosuppressive therapy had received pneumococcal booster vaccinations between M12 and M36. PWH had had a mean CD4 count of 588.6 cells/mm^3^, were all on combination antiretroviral therapy (cART), 2 had had an AIDS diagnosis, and 89% (49/55) had been virologically suppressed (HIV viral load <20 copies/mL) (Table 1). Among patients on immunosuppressive therapy, 31 had been on cIM, 43 on bIM, 43 on combination therapy, and 14 had been in the switched group. Inflammatory bowel disease was the most common diagnosis in patients on cIM, bIM and the switched group (32%–79%), while a kidney transplantation was most common in patients on combination therapy (37%) (Table 2).

**Table 1.** Baseline Characteristics of Study Participants

	Total (N = 205)	PWH (n = 55)	Patients on Immunosuppressive Therapy (n = 131)	Controls (n = 19)	*P V*alue Across Groups
Males, n (%)	116/205 (57%)	46/55 (84%)^a^	62/131 (47%)*	8/19 (42%)*	**<.001**
Age, median (IQR)	47 (32–56)	51 (43–59)^a^	44 (30–56)*	50 (34–54)	**.009**
BMI kg/m^2^, median (IQR)	24.1 (21.9–27.0)	23.7 (22.0–25.9)	24.2 (22.0–27.3)	23.7 (21.3–28.7)	.689
Charlson comorbidity index, median (IQR)	1 (1–2)	1 (0–2)	1 (1–2)	2 (1–2)	.003
Chronic kidney disease (eGFR <60), n (%)	36/205 (18%)	11/55 (20%)	24/131 (18%)	1/19 (5%)	.366
Past or current smoker, n (%)	92/205 (45%)	35/55 (64%)^a^	46/131 (35%)*	11/19 (58%)	**<.001**
Heavy alcohol use (>7 units/wk), n (%)	43/205 (21%)	13/55 (24%)	27/131 (21%)	3/19 (16%)	0.749
Illicit drug use, n (%)	28/205 (14%)	16/55 (29%)^a^	11/131 (8%)*	1/19 (5%)*	**<.001**
PCV13 booster dose at 24 m, n (%)‡	9/205 (4%)	3/55 (5%)	6/131 (5%)	0/19 (0%)	.363
PPSV23 booster dose at 24 m, n (%)‡	9/205 (4%)	5/55 (9%)	4/131 (13%)	0/19 (0%)	.166
HIV-specific	NA	…	NA	NA	NA
Time since HIV diagnosis, y (median, IQR)	NA	9 (4–18)	NA	NA	NA
AIDS at time of diagnosis, n (%)	NA	2/56 (4%)	NA	NA	NA
cART use, n (%)	NA	56/56 (100%)	NA	NA	NA
Undetectable viral load, n (%)	NA	49/55 (89%)	NA	NA	NA
Current CD4 count cells/mm^3^ (mean, SD)	NA	588.6 (277.3)	NA	NA	NA
Current CD4 count <500 cell/mm^3^, n (%)	NA	24/55 (44%)	NA	NA	NA
Nadir CD4 count cells/mm^3^ (mean, SD)^b^	NA	268.2 (169.7)	NA	NA	NA
Nadir CD4 count <200 cells/mm^3^, n (%)^b^	NA	17/46 (37%)	NA	NA	NA
CD4/CD8 ratio n (mean, SD)	NA	0.817 (0.421)	NA	NA	NA

Significant *P* values are in bold. *P* values for dichotomous data were calculated by χ^2^, or by Fisher's Exact Test when the expected counts in any of the cells were below 5; *P* values of normally distributed numerical data were calculated by one-way analysis of variance (ANOVA), and non-normally distributed data by Kruskal–Wallis test (data not normally distributed).

Abbreviations: BMI, body mass index; cART, combination antiretroviral therapy; eGFR, estimated glomerular filtration rate; PCV13, 13-valent pneumococcal conjugate vaccine (Prevenar 13^®^); PWH, people with human immunodeficiency virus (HIV); PPSV23, 23-valent polysaccharide vaccine (Pneumovax 23^®^).

^a^Is significantly higher than * ‡ Among PWH, 3 received PCV13 + PPSV23 and 2 PPSV23 only; among patients on immunosuppressive therapy 4 received PCV13 + PPSV23 and 2 PCV13 only.

^b^Nadir CD4 count is missing in 9 participants.

**Table 2.** Baseline Characteristics of Patients on Immunosuppressive Therapy

	Total (N = 131)	Conventional Immunomodulators (n = 31)	Biological Immunomodulators (n = 43)	Combination Therapy (n = 43)	“Switched” (n = 14)
Underlying disease					
Inflammatory bowel disease, n (%)	53 (40%)	10 (32%)	18 (42%)	14 (33%)	11 (79%)
Rheumatoid arthritis, n (%)	16 (12%)	6 (19%)	3 (7%)	7 (16%)	0 (0%)
Psoriasis/psoriatic arthritis, n (%)	20 (15%)	3 (10%)	10 (23%)	4 (9%)	3 (21%)
Kidney transplantation, n (%)	16 (12%)	0 (0%)	0 (0%)	16 (37%)	0 (0%)
Other diagnosis, n (%)^a^	26 (20%)	12 (40%)	12 (24%)	2 (5%)	0 (0%)
Conventional immunomodulators					
Methotrexate (7.5–25 mg/wk), n (%)	28 (21%)	13 (42%)	NA	14 (33%)	1 (7%)
Prednisolone (>10 mg/day or 700 mg cumulative), n (%)	9 (7%)	1 (3%)	NA	7 (16%)	1 (7%)
Azathioprine, n (%)	14 (11%)	8 (26%)	NA	5 (12%)	1 (7%)
Mercaptopurine, n (%)	9 (7%)	3 (10%)	NA	3 (7%)	3 (21%)
Thioguanine, n (%)	3 (2%)	1 (3%)	NA	2 (5%)	0 (0%)
Tacrolimus, n (%)	11 (8%)	1 (3%)	NA	10 (23%)	0 (0%)
Cyclosporine, n (%)	2 (2%)	0 (0%)	NA	2 (5%)	0 (0%)
Mycophenolate mofetil, n (%)	12 (9%)	3 (10%)	NA	9 (21%)	0 (0%)
Mycofenol acid (Myfortic), n (%)	2 (2%)	0 (0%)	NA	2 (5%)	0 (0%)
Other conventional immunomodulator, n (%)^b^	3 (2%)	1 (3%)	NA	2 (5%)	0 (0%)
Biological immunomodulators					
Adalimumab, n (%)	29 (22%)	NA	15 (35%)	7 (16%)	7 (50%)
Infliximab, n (%)	20 (15%)	NA	9 (21%)	9 (21%)	2 (14%)
Etanercept, n (%)	6 (5%)	NA	4 (9%)	2 (5%)	0 (0%)
Rituximab, n (%)	4 (3%)	NA	2 (5%)	2 (5%)	0 (0%)
Ocrelizumab, n (%)	3 (2%)	NA	3 (7%)	0 (0%)	0 (0%)
Tofacitinib, n (%)	3 (2%)	NA	2 (5%)	1 (2%)	0 (0%)
Ustekinumab, n (%)	7 (5%)	NA	3 (7%)	3 (7%)	1 (7%)
Other biological immunomodulator, n (%)^c^	6 (5%)	NA	5 (12%)	1 (2%)	0 (0%)

^a^Other diagnosis: sarcoidosis (n = 3); neurological autoimmune disease (n = 3); systemic lupus erythematosus (SLE) (n = 2); systemic vasculitis (n = 2); M. Bechterew (n = 1); SLE (n = 1); atopic eczema (n = 1); autoimmune hepatitis (n = 1); antisynthetase syndrome (n = 1); juvenile idiopathic arthritis (n = 1); juvenile idiopathic arthritis and uveitis anterior (n = 1); Morbus Behcet (n = 1); Morbus Sjögren (n = 1); membranous glomerulonephritis (n = 1); mixed connective tissue disease (n = 1); multiple sclerosis (n = 1); spondylarthritis (n = 1); uveitis and papillitis (n = 1); systemic disease with features of Morbus Sjögren (n = 1).

^b^Other conventional immunomodulators: sirolimus (n = 1); cyclophosphamide (n = 1); hydroxyurea (n = 1).

^c^Other biological immunomodulator: golimumab (n = 1); certolizumab (n = 1); tocilizumab (n = 1); natalizumab (n = 1); dupilumab (n = 1); ixekizumab (n = 1).

### Serological Response Rates

**Figure 1.**
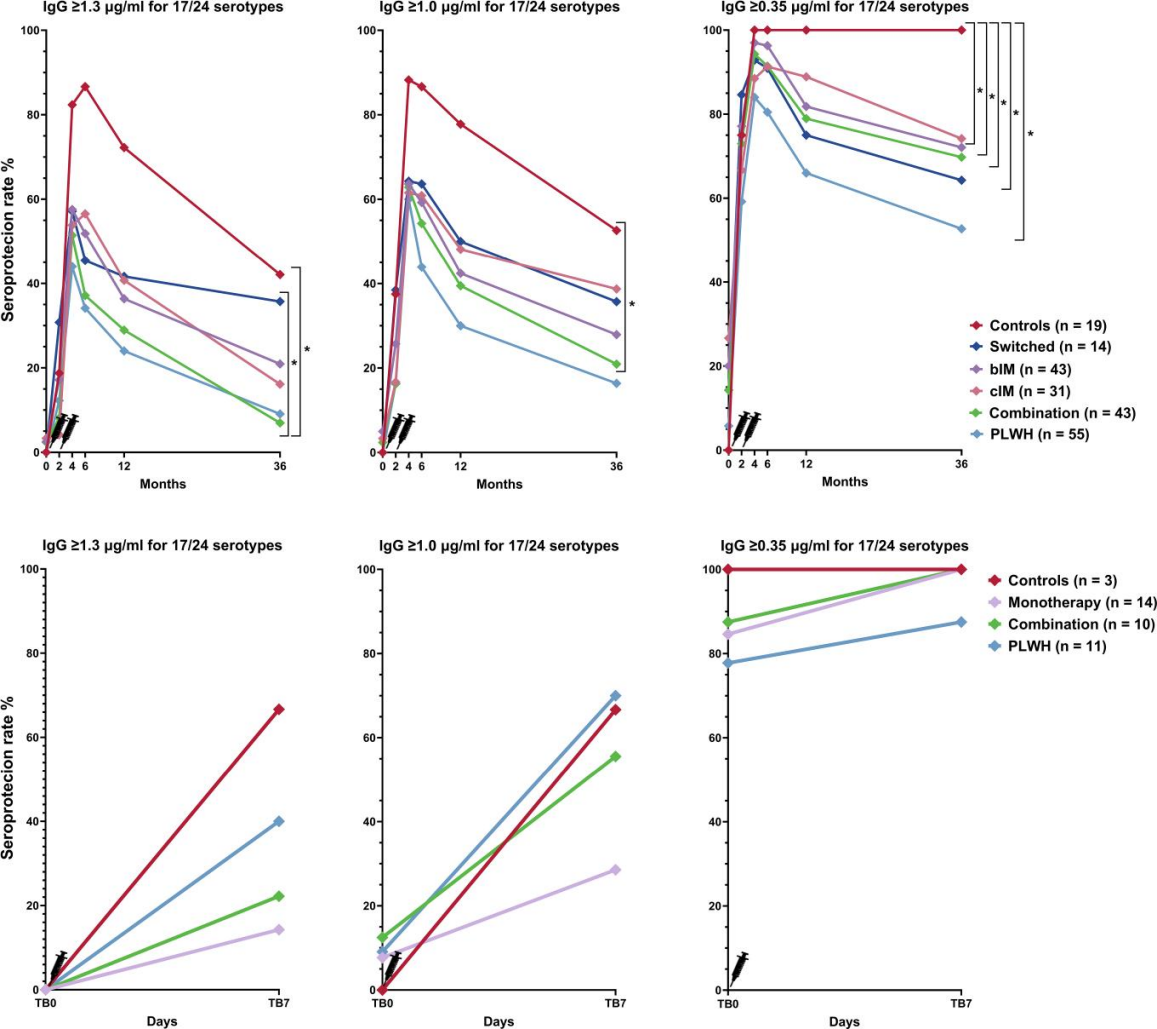
Seroprotection rates over time and rapid recall responses in patients on immunosuppressive therapy, people with human immunodeficiency virus (PWH), and controls, shown for different cutoff of seroprotection. The figures on the top display serological responses among patients on biological immunomodulators (bIM), patients on conventional immunomodulators (cIM), patients on combination therapy, patients in the switched group, PWH and controls (bottom), who received PCV13 followed by PPSV23 1 months later. *Significant difference in serological response rate at M36 between groups as calculated by the χ^2^ test. The figures on the bottom display rapid recall responses among patients on biological immunomodulators (bIM), patients on conventional immunomodulators (cIM), patients on combination therapy, patients in the switched group, and controls (top), and PWH and controls (bottom), who received a PCV20 booster. No significant differences in serological response rates at DB7 were observed between groups as calculated by the χ^2^ test.

SPRs significantly dropped between M4 to M36 from 40% (22/50) to 9% (5/55) in PWH, and from 45% (59/108) to 17% (22/131) in patients on immunosuppressive therapy; whereas in controls, the SPR dropped from 82% (14/17) to 42% (8/19). Declines in SPR did not significantly differ between groups (*P* value = .31). Results remained similar when participants who had received booster doses or anti-CD20 therapy were excluded (Supplementary Table 1). Moderate serological responses (≥0.35 µg/mL) at M36 were observed in 53% (29/55) of PWH, 71% (93/131) and 100% (17/17) of controls. At M36, serological responses were significantly lower in PWH and patients on combination therapy compared with controls, regardless of the correlate of protection applied (Figure 1; Supplementary Table 2). Among patients on immunosuppressive therapy, those on combination therapy had significantly lower SPRs than the switched group (7% vs 36%, *P* = .043) (Supplementary Table 3). SPRs for PCV13 serotypes were significantly lower for PWH (13% [7/55]) and patients on immunosuppressive therapy (22% [29/131]) compared with controls (53% [10/19]) (*P* value = .002). SPRs for PPSV23-unique serotypes were numerically lower in PWH (33% [18/55]) and patients on immunosuppressive therapy (44% [58/131]) than in controls (63% [12/19]), although this difference was not statistically different (*P* value = .061; Supplementary Table 4).

### Antibody Concentrations

**Figure 2.**
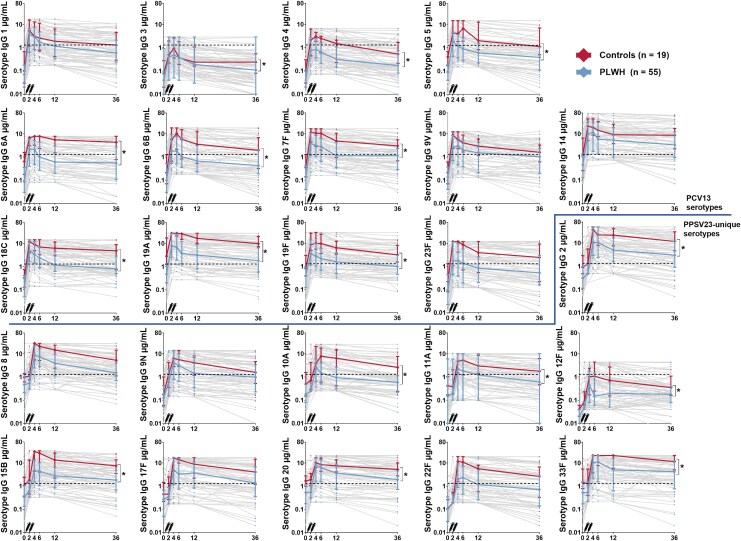
Serotype-specific IgG concentrations for all 24 serotypes in people with human immunodeficiency virus (PWH) and controls. The figures display serotype-specific serological responses PWH and controls, who received PCV13 followed by PPSV23 1 month later. The symbols depict median IgG concentrations and interquartile ranges. The gray lines depict IgG concentrations of individual participants. The dotted line indicates an IgG level of 1.3 µg/mL. * Significant difference in serotypes-specific IgG level at M36 between PWH and controls as calculated by the independent samples Mann–Whitney *U* test. The dotted line indicates an IgG level of 1.3 µg/mL.

**Figure 3.**
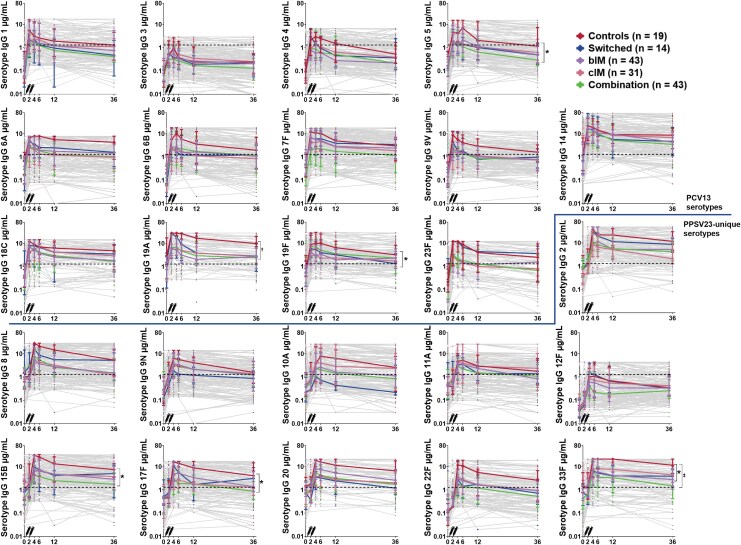
Serotype-specific antibody concentrations for all 24 serotypes in patients on immunosuppressive drugs and controls. The figures display serotype-specific serological responses among patients on biological immunomodulators (bIM), patients on conventional immunomodulators (cIM), patients on combination therapy, patients in the switched group, and controls, who received PCV13 followed by PPSV23 1 months later. The symbols depict median IgG concentrations and interquartile ranges. The gray lines depict IgG concentrations of individual participants. The dotted line indicates an IgG level of 1.3 µg/mL. *Significant difference in antibody level at M36 between patients on combination therapy and controls as calculated by independent samples Kruskal–Wallis test. †Significant difference in antibody level at M36 between patients on conventional immunomodulators, patients on biological immunomodulators, patients on combination therapy and the switched group in comparison to controls, as calculated by independent samples Kruskal–Wallis test. ‡Significant difference in antibody level at M36 between patients on biological immunomodulators and controls calculated by independent samples Kruskal–Wallis test.

Median serotype-specific IgG concentrations dropped below the protective cutoff (1.3 μg/mL) at M36, for 16/24 (67%) of serotypes in PWH, 12/24 (50%) in patients on immunosuppressive therapy and 5/24 (20%) in controls. Compared with controls, median IgG concentration were significantly lower for PWH for 9/13 (69%) PCV13 serotypes and for 7/11 (64%) PPSV23-unique serotypes (Figure 2). Median IgG concentrations were significantly lower in patients on immunosuppressive combination therapy compared with controls for 3/13 (23%) PCV13 serotypes, and for 3/11 (27%) PPSV23-unique serotypes (Figure 3). Median fold antibody changes between M4 and M36 ranged 0.24–0.50 in PWH, 0.37–0.76 in patients on immunosuppressive therapy and 0.29–0.87 in controls. Declines were significantly higher for PWH than controls for serotypes 5, 17F and 33F; changes in patients on immunosuppressive therapy were comparable to controls (Supplementary Table 5). Fold changes were comparable across immunosuppressant subgroups, except for serotype 10A, with a larger decline for cIM than bIM (0.58 vs 0.27, *P* value = .017) (Supplementary Table 6).

### Predictors of Seroprotection

No logistic regression analysis was performed for PWH as only 5 participants were seroprotected at M36; instead, serological responses were stratified by relevant variables. Both patients with AIDS at initial study enrolment and all 6 patients with a detectable viral load showed poor serological responses at M36 (Supplementary Table 7). For patients on immunosuppressive therapy, univariable analysis found a negative association between combination therapy use and seroprotection at M36 (OR 0.27 [95% CI .08–.98]) and a positive association between seroprotection at M4 (OR 16.36 [95% CI 2.08–129.05]) and seroprotection at M36. In the multivariable model, significant positive predictors for seroprotection at M36 were being in the switched group (aOR 5.25 [95% CI 1.23–22.51]) and seroprotection at M4 (aOR 18.40 [95% CI 2.23–151.68]) (Supplementary Table 8). Among participants with a good serological response at M4, 96% (91/95) retained at least a moderate response at M36 (Supplementary Table 9).

### Immunological Memory

Of those with a good response at M4, 72% (68/95) had sero-reverted at M36, including 17 PWH, 25 patients on immunosuppressive monotherapy, 16 patients on immunosuppressive combination therapy and 7 controls. Of those, 11 PWH, 14 patients on immunosuppressive monotherapy, 10 patients on immunosuppressive combination therapy and 3 controls agreed to receive a PCV20 booster at a median of 5 years (IQR 56–63 months) post-PCV13. Rapid recall responses (good serological responses for ≥70% of serotypes included in PCV13/PCV20/PPSV23) were observed in 40% (4/10) of PWH, 14% (2/14) patients on immunosuppressive monotherapy, 22% (2/9) patients on combination therapy, and 67% (2/3) controls. All but 1 participant, a 65-year-old PWH, showed at least a moderate serological response at DB7 (Figure 1). Protection rates at DB7 were similar for PCV13 and PPSV23-unique serotypes (Supplementary Table 10). Significant antibody increases between DB0 and DB7 were observed for 7/13 (54%) PCV13/PCV20-shared serotypes, but for none of the 7 PPSV23/PCV20-shared serotypes (Supplementary Table 11). No notable trends were observed when stratifying serological responses at DB7 by relevant variables (Supplementary Table 12).

## DISCUSSION

Three years postvaccination, SPRs had declined markedly in all groups, with particularly low persistence observed among ICPs. The decline in ICPs was similar to controls. SPRs were significantly lower in PWH and patients on immunosuppressive therapy compared with controls, consistent with findings 2 months postvaccination. No prior studies reported on the long-term serological responses of the combined PCV13/PPSV23 schedule, but several studies investigating short-term immunogenicity showed similar findings [5, 6, 18, 19]. A meta-analysis displayed lower serological responses 1–3 month after PCV13/PPSV23 in PWH compared with controls [18]. Also, SPRs were lower 4–8 weeks after PCV13/PPSV23 in IBD patients on immunosuppressive therapy compared with those without [20]. Among PWH, those with AIDS (n = 2) or detectable HIV viral load at vaccination (n = 6) all showed poor responses at M36, consistent with prior studies on unsuppressed HIV-infections [21, 22]. Among patients on immunosuppressive drugs, use of combination therapy was a significant predictor for lower SPRs 3 years post-PCV13/PPSV23. Prior research on IBD patients also observed more severely impaired immune responses among those on combination therapy [20], a phenomenon also observed with hepatitis A vaccination [23].

PCV13 contains pneumococcal polysaccharides conjugated to the CRM197 protein, which elicits a T-cell-dependent immune response by engaging T-helper cells, typically resulting in stronger immunological memory. In contrast, PPSV23 induces a T-cell-independent response through direct B-cell activation by polysaccharides. Since most ICPs in our study had T-cell deficiencies, a larger impairment in response to the T-cell-dependent PCV13 compared with PPSV23 was anticipated. However, immune responses measured by SPR and median serotype-specific IgG concentrations were similarly reduced in ICPs for both PCV13 and PPSV23-unique serotypes. We assessed the immunological memory 5 years post-PCV13/PPSV23 by measuring rapid recall responses after a PCV20 booster among a small subset who had sero-reverted. Rapid recall responses were observed in 40% of PWH, 17% of patients on immunosuppressive therapy compared with 67% of controls. Significant increases in antibody levels were observed for 54% of shared PCV13/PCV20 serotypes, but not for PPSV23/PCV20-shared serotypes. This indicates that PWH and patients on immunosuppressive therapy who initially responded to vaccination can mount PCV-specific immunological memory, whereas PPSV23 does not induce boostable memory. This aligns with a prior study in IBD patients showing a 5-fold reduction in severe IPD risk among those who received PCV, while PPSV23 had no significant effect [24]. Notably, rapid recall responses after PCV20 were higher in a cohort of allogeneic hematopoietic progenitor cell transplantation (allo-HCT) recipients who had received a 3-dose PCV13 priming regimen 3–6 months post-allo-HCT, followed by PCV13 and PPSV23 boosters [25]. Rapid recall responses were observed in 82% of allo-HCT recipients, whereas this was 40% for PWH and 14%–22% for patients on immunosuppressive therapy, as reported here. As allo-HCT recipients are generally more severely immunocompromised, it is likely that the expanded PCV13-priming regimen they received established a more robust immunological memory. Thus, we expect that an expanded PCV-regimen may also enhance the durability of immunity among PWH and patients on immunosuppressive therapy.

### Strengths and Limitations

To our knowledge, this is the first study to assess long-term PCV/PPSV23 immunogenicity and PCV20 immunogenicity in PWH and patients on immunosuppressive therapy. One limitation was that only participants from the initial study were eligible, reducing the sample size. Notably, only 3 of 7 controls who sero-reverted agreed to recall responses assessments, limiting the ability to detect differences between ICPs and controls. As such, subgroup analyses and secondary outcomes should be interpreted as exploratory, as the study was not powered for these comparisons. Additionally, missing baseline data for participants lost to follow-up may have introduced selection bias. Another limitation was the use of correlates of immunity to assess long-term protection, as large sample sizes needed for clinical efficacy trials [26] are unrealistic for PWH and patients on immunosuppressive therapy. The next-best proxy is measuring correlates of immunity, although in adults, these are ill-defined [19]. The AAAAI's conservative cutoff of ≥1.3 µg/mL has been widely used, but previous studies have applied less stringent definitions, complicating the generalizability of findings [12]. The AAAAI definition is based on minimal protective antibody levels determined in 1998 using radioimmunoassay of sera from individuals who developed pneumococcal disease despite vaccination [27], resulting in a cutoff of ≥1.3 µg/mL [28]. This threshold has since been used in multiple studies involving both ICPs and immunocompetent adults [10, 11, 20]. The WHO suggests a lower threshold of ≥0.35 µg/mL, derived from PCV7 efficacy trials in infants [29]. However, Andrews et al later showed that this cutoff does not correlate well with protection across all PCV13 serotypes, suggesting ≥1.0 µg/mL may be more appropriate [13]. To improve comparability with prior studies, we presented our results using all 3 cutoffs [12–14]. As a result, estimates of immunogenicity and boostability depend on the chosen correlate and may be underestimated or overestimated, an ongoing challenge in pneumococcal vaccine efficacy evaluations. Additionally, we only assessed humoral responses, even though T-cell-mediated B-cell memory also plays a key role in long-term protection against IPD. The presence of rapid recall responses after a PCV20 booster was used as a proxy for immunological memory.

### Recommendation for Clinical Practice and Future Research

Current CDC guidelines recommend a single PCV20-dose for ICPs without prior pneumococcal vaccination or ≥5 years after PCV13/PPSV23 [6]. Our findings underscore that good serological responses persist only in a minority of PWH and patients on immunosuppressive therapy, and in less than half of immunocompetent controls, but that a PCV20 booster 5 years after priming with PCV13/PPSV23 could evoke recall responses for PCV-serotypes in a proportion of those who had sero-reverted. As no recall responses were observed for shared PPSV23/PCV20-serotypes, this supports discontinuing PPSV23 in favor of highly valent PCVs. To enhance initial serological priming, multiple instead of single PCV-doses may be necessary, but more research on this topic is needed, especially among those with T-cell deficiencies, such as PWH and patients with medication impairing T-helper cells. Bhorat et al found moderately increased antibody levels after 2 additional subsequent PCV13-doses among PWH [30]. Additionally, a 5-year follow-up study showed slightly better outcomes for 2 PCV13-doses versus a single PPSV23-dose [31]. However, neither study assessed memory responses. Future studies should focus on both immunogenicity and memory responses of expanded priming schedules of PCV20 and other higher-valent PCV candidates, such as V114, which includes 17 serotypes complementary to PCV20 [32].

### Conclusions

Our findings indicate that PPSV23 should be replaced by highly valent PCVs. For both vaccines, good serological responses persisted in only a minority of PWH and patients on immunosuppressive therapy, and less than half of immunocompetent individuals. However, 5 years postvaccination, only PCV serotypes, and not PPSV23-unique serotypes, were boostable by PCV20, indicating the presence of immunological memory. Future research must elucidate whether expanded priming schedules of higher-valent PCVs, such as PCV20, can improve durability of protection.

## Supplementary Material

ciaf438_Supplementary_Data

## Supplementary Data

Supplementary materials are available at *Clinical Infectious Diseases* online. Consisting of data provided by the authors to benefit the reader, the posted materials are not copyedited and are the sole responsibility of the authors, so questions or comments should be addressed to the corresponding author.
