# Supplementary material for: Long-term Immunogenicity and Boostability of the 13-valent Pneumococcal Conjugate Vaccine Followed by the 23-valent Pneumococcal Polysaccharide Vaccine in Adults Receiving Immunosuppressive Therapy and Adults With HIV—3-year Follow-up of a Prospective Cohort Study
Source: Clin Infect Dis. 2025 Aug 6;81(5):e432–40. doi: 10.1093/cid/ciaf438 (PMC12728288; doi:10.1093/cid/ciaf438)
Supplement: ciaf438_Supplementary_Data [file ciaf438_supplementary_data.docx]

**Supplementary Material**

**Table of contents**

[**Supplementary Table 1.** Seroprotection rates at M36 in all participants with and without booster. 2](#_Toc204244196)

[**Supplementary Table 2**. Seroprotection rates at M4 and M36. 3](#_Toc204244197)

[**Supplementary Table 3.** Seroprotection rates in patients on immunosuppressive drugs. 4](#_Toc204244198)

[**Supplementary Table 4.** PCV13 and PPSV23-unique serotype-specific seroprotection rates at M36. 5](#_Toc204244199)

[**Supplementary Table 5.** Changes in serotype-specific antibody concentrations between M4 and M36. 6](#_Toc204244200)

[**Supplementary Table 6.** Changes in serotype-specific antibody concentrations between M4 and M36 in patients on immunosuppressive therapy. 10](#_Toc204244201)

[S**upplementary Table 7**. Serologic response on M36 stratified by relevant variables among PLWH. 16](#_Toc204244202)

[S**upplementary Table 8**. Factors associated with seroprotection at M36. 18](#_Toc204244203)

[S**upplementary Table 9.** Serological responses at M36 among those with a good serological response at M36. 19](#_Toc204244204)

[S**upplementary Table 10.** PCV13 and PPSV23-unique serotype-specific protection rates at DB7. 20](#_Toc204244205)

[**Supplementary Table 11.** Serotype-specific antibody concentrations at DB0 and DB7. 21](#_Toc204244206)

[**Supplementary Table 12.** Rapid recall responses stratified by relevant variables. 25](#_Toc204244207)

[S**upplementary Figure 1.** Study procedures. 27](#_Toc204244208)

[S**upplementary Figure 2.** Flowchart of study participants. 28](#_Toc204244209)

Supplementary Table 1. Seroprotection rates at M36 in all participants with and without booster.

| Groups | Overall  (% [95%CI]) | Without booster (% [95%CI]) | With booster* (% [95%CI]) | Without anti-CD20 therapy  (% [95%CI]) | With anti-CD20 therapy  (% [95%CI]) |
| --- | --- | --- | --- | --- | --- |
| PLWH | 5/55 (9% [4-19%]) | 5/50 (10% [4-21%]) | 0/5 (0% [0-38%]) | NA | NA |
| Patients on immunosuppressive therapy | 22/131 (17% [11-24%]) | 21/125 (17% [11-24%]) | 1/6 (17% [2-56%]) | 22/127 (17% [12-25%]) | 0/4 (0% [0-45%]) |
| Controls | 8/19 (42% [22-64%]) | 8/19 (42% [22-64%]) | NA | NA | NA |

*Among PLWH, three received PCV13 + PPSV23, and two PPSV23 only; among patients on immunosuppressive therapy four received PCV13 + PPSV23 and two PCV13 only. PLWH = people living with HIV; NA = not applicable.

## **Supplementary Table 2**. Seroprotection rates at M4 and M36.

| Timepoint | PLWH | Immunosuppressive therapy | Controls | *p*-value difference between groups |
| --- | --- | --- | --- | --- |
| M4 | 22/50 (44%)* | 59/108 (55%)* | 14/17 (82%)† | **0.023** |
| M36 | 5/55 (9%)* | 22/131 (17%)* | 8/19 (42%)† | **0.004** |
| *p*-value difference between timepoints | **<0.001** | **<0.001** | 0.109 | 0.306‡ |

Significant *p*-values are in **bold**. *P*-values that show differences in proportions between groups are calculated by the Chi-square test; *P*-values that show differences between timepoints are calculated by the McNemar test. *is significantly higher than †. ‡ This p-value shows difference in participants losing protection between groups and was calculated with the Chi-square test. PLWH = people living with HIV.

## **Supplementary Table 3.** Seroprotection rates in patients on immunosuppressive drugs.

| Timepoint | bIM | cIM | Combination therapy | Switched group | *p*-value difference between groups |
| --- | --- | --- | --- | --- | --- |
| M4 | 19/33 (58%) | 14/26 (54%) | 18/35 (51%) | 8/14 (57%) | ns |
| M36 | 9/43 (21%) | 5/31 (16%) | 3/43 (7%)† | 5/14 (36%)* | 0.069 |
| *p*-value difference between timepoints | **<0.001** | **0.002** | **<0.001** | 0.250 |  |

Significant *p*-values are in **bold**. *P*-values show differences in proportions between groups and are calculated by the Chi-square test; *P*-values that show differences between timepoints are calculated by the McNemar test. *is significantly higher than †. bIM = biological immunomodulator; cIM = conventional immunomodulator.

## **Supplementary Table 4.** PCV13 and PPSV23-unique serotype-specific seroprotection rates at M36.

| Groups | Above cut-off for 9/13 (70%) of PCV13-serotypes | | | Above cut-off for 8/11 (70%) of PPSV23-unique serotypes | | |
| --- | --- | --- | --- | --- | --- | --- |
|  | **> 1.3 µg/mL** | **> 1.0 µg/mL** | **> 0.35 µg/mL** | **> 1.3 µg/mL** | **> 1.0 µg/mL** | **> 0.35 µg/mL** |
| PLWH | 7/55 (13%)†§ | 10/55 (18%)† | 29/55 (53%)† | 18/55 (33%) | 22/55 (40%)† | 41/55 (75%)† |
| Patients on bIM | 11/43 (26%)† | 16/43 (37%) | 31/43 (72%) | 21/43 (49%) | 26/43 (60%)* | 38/43 (88%) |
| Patients on cIM | 9/31 (29%) | 14/31 (45%)* | 23/31 (74%) | 13/31 (42%) | 20/31 (65%)* | 27/31 (87%) |
| Patients on combination therapy | 4/43 (9%)†§ | 10/43 (23%) | 26/43 (60%) | 17/43 (40%) | 21/43 (49%)† | 34/43 (79%) |
| Switched group | 5/14 (36%)‡ | 5/14 (36%) | 12/14 (86%)* | 7/14 (50%) | 7/14 (50%) | 10/14 (17%) |
| Controls | 10/19 (53%)* | 10/19 (53%)* | 17/19 (89%)* | 12/19 (63%) | 15/19 (79%)* | 19/19 (100%)* |
| *p*-value | **0.002** | **0.012** | **0.007** | 0.061 | **0.009** | **0.039** |

Significant *p*-values are in **bold**. * is significantly higher than †. ‡ is significantly higher than §. *P*-values show differences in proportions between groups and are calculated by the Chi-square test. PLWH = people living with HIV; bIM = biological immunomodulator; cIM = conventional immunomodulator.

## **Supplementary Table 5.** Changes in serotype-specific antibody concentrations between M4 and M36.

|  | PLWH | | | Immunosuppressive therapy | | | Controls | | |  |
| --- | --- | --- | --- | --- | --- | --- | --- | --- | --- | --- |
| Serotype | **M4** | **M36** | **Median fold change** | **M4** | **M36** | **Median fold change** | **M4** | **M36** | **Median fold change** | ***p*-value** |
| IgG 1 | 2.95 (0.17-17.00) | 0.59 (0.02-10.73) | 0.28 (0.06-1.69)† | 1.95 (0.17-17.00) | 0.58 (0.02-17.00) | 0.39 (0.06-10.40)* | 3.08 (0.17-17.00) | 1.29 (0.03-10.55) | 0.35 (0.16-0.62) | **0.036** |
| IgG 2 | 10.68 (0.48-49.26) | 3.00 (0.05-49.26) | 0.32 (0.01-4.14)† | 10.79 (0.48-49.26) | 4.53 (0.08-49.26) | 0.51 (0.03-17.22)* | 37.65 (0.89-49.26) | 11.77 (0.24-49.26) | 0.38 (0.10-34.26) | **0.007** |
| IgG 3 | 0.48 (0.05-2.90) | 0.11 (0.01-2.90) | 0.24 (0.01-1.04)† | 0.46 (0.03-2.90) | 0.16 (0.01-2.90) | 0.39 (0.05-5.66)* | 0.94 (0.08-2.90) | 0.24 (0.01-2.79) | 0.30 (0.10-0.96) | **0.001** |
| IgG 4 | 0.83 (0.07-6.66) | 0.17 (0.01-5.43) | 0.28 (0.00-1.51) | 1.06 (0.07-6.66) | 0.27 (0.01-6.66) | 0.38 (0.02-19.13) | 3.00 (0.10-6.66) | 0.53 (0.04-6.66) | 0.36 (0.07-1.56) | 0.124 |
| IgG 5 | 1.22 (0.15-15.02) | 0.39 (0.01-15.02) | 0.26 (0.07-1.00)† | 1.62 (0.15-15.02) | 0.46 (0.02-15.02) | 0.40 (0.04-9.50)* | 4.14 (0.25-15.02) | 1.15 (0.11-15.02) | 0.39 (0.13-1.00)* | **<0.001** |
| IgG 6A | 2.12 (0.08-7.86) | 0.55 (0.01-7.86) | 0.36 (0.05-1.68) | 2.60 (0.08-7.86) | 1.12 (0.02-7.86) | 0.48 (0.06-41.71) | 7.86 (1.88-7.86) | 4.32 (0.27-7.86) | 0.62 (0.12-1.00) | **0.033** |
| IgG 6B | 1.80 (0.18-18.10) | 0.43 (0.02-13.04) | 0.36 (0.02-1.05) | 2.47 (0.18-18.10) | 1.06 (0.02-18.10) | 0.37 (0.06-5.84) | 10.37 (0.18-18.10) | 1.95 (0.06-18.10) | 0.41 (0.10-1.98) | 0.249 |
| IgG 7F | 3.15 (0.16-16.60) | 1.14 (0.03-11.52) | 0.32 (0.00-0.81) | 5.13 (0.16-16.60) | 1.87 (0.04-16.60) | 0.41 (0.04-10.97) | 10.64 (1.35-16.60) | 3.03 (0.49-16.60) | 0.37 (0.16-1.00) | 0.057 |
| IgG 8 | 8.60 (0.31-28.48) | 1.52 (0.07-28.48) | 0.29 (0.02-1.00)† | 7.20 (0.28-28.48) | 1.79 (0.05-28.48) | 0.49 (0.06-3.88)* | 28.48 (0.85-28.48) | 5.33 (0.54-28.48) | 0.31 (0.08-7.70) | **<0.001** |
| IgG 9N | 3.99 (0.14-14.06) | 0.99 (0.01-14.06) | 0.32 (0.01-1.26) | 3.54 (0.14-14.06) | 1.26 (0.03-14.06) | 0.42 (0.03-5.04) | 6.38 (0.36-14.06) | 1.59 (0.13-14.06) | 0.29 (0.10-2.09) | **0.035** |
| IgG 9V | 3.00 (0.13-12.88) | 1.03 (0.01-12.88) | 0.31 (0.01-4.44)† | 1.78 (0.13-12.88) | 0.77 (0.02-12.88) | 0.46 (0.05-6.16)* | 5.62 (0.24-12.88) | 1.61 (0.22-9.85) | 0.35 (0.05-0.89) | **0.005** |
| IgG 10A | 2.63 (0.25-25.96) | 0.59 (0.03-25.96) | 0.26 (0.03-1.00)† | 2.34 (0.25-25.96) | 0.87 (0.03-25.96) | 0.44 (0.00-5.92)* | 3.89 (0.27-25.96) | 2.59 (0.12-16.44) | 0.37 (0.06-20.70) | **<0.001** |
| IgG 11A | 2.18 (0.10-10.16) | 0.61 (0.01-10.16) | 0.29 (0.02-1.20)† | 3.21 (0.10-10.16) | 1.36 (0.03-10.16) | 0.54 (0.06-98.00)* | 4.81 (0.10-10.16) | 1.79 (0.01-10.16) | 0.40 (0.15-5.49) | **<0.001** |
| IgG 12F | 0.45 (0.04-4.42) | 0.18 (0.00-4.42) | 0.43 (0.04-8.66)† | 0.61 (0.04-4.42) | 0.30 (0.02-4.42) | 0.76 (0.02-85.78)* | 1.03 (0.05-4.42) | 0.35 (0.12-4.42) | 0.48 (0.08-11.29) | **0.030** |
| IgG 14 | 11.59 (0.74-75.98) | 3.35 (0.07-69.64) | 0.45 (0.02-1.58) | 12.81 (0.74-75.98) | 4.61 (0.07-75.98) | 0.42 (0.05-32.27) | 18.83 (0.74-75.98) | 8.71 (0.07-61.92) | 0.43 (0.10-1.40) | 0.922 |
| IgG 15B | 4.77 (0.33-33.88) | 1.83 (0.05-20.05) | 0.29 (0.07-5.51)† | 6.2 (0.33-33.88) | 2.90 (0.03-33.88) | 0.50 (0.00-31.91)* | 31.00 (0.88-33.88) | 7.53 (0.51-25.57) | 0.48 (0.19-15.54) | 0.**002** |
| IgG 17F | 5.09 (0.17-17.02) | 1.38 (0.02-17.02) | 0.36 (0.02-10.87)† | 3.54 (0.17-17.02) | 1.30 (0.02-17.02) | 0.41 (0.04-102.40) | 16.33 (0.20-17.02) | 4.10 (0.04-17.02) | 0.54 (0.09-8.95)* | **0.016** |
| IgG 18C | 3.33 (0.14-14.60) | 0.81 (0.05-7.86) | 0.36 (0.05-1.32)† | 6.1 (0.14-14.60) | 2.29 (0.05-14.60) | 0.55 (0.03-3.05)* | 9.07 (2.29-14.60) | 4.84 (0.23-14.60) | 0.44 (0.04-2.31) | **0.004** |
| IgG 19A | 6.97 (0.27-27.74) | 1.84 (0.04-27.74) | 0.37 (0.06-1.00) | 6.20 (0.27-27.74) | 2.64 (0.10-27.74) | 0.44 (0.05-7.75) | 27.74 (9.19-27.74) | 9.84 (0.60-27.74) | 0.51 (0.26-1.00) | **0.031** |
| IgG 19F | 2.77 (0.41-29.22) | 0.99 (0.08-29.22) | 0.36 (0.10-1.00) | 4.23 (0.29-29.22) | 2.00 (0.08-28.12) | 0.41 (0.02-4.25) | 9.81 (2.29-29.22) | 3.16 (0.92-13.69) | 0.35 (0.07-1.10) | 0.351 |
| IgG 20 | 8.03 (0.20-20.94) | 1.93 (0.02-20.94) | 0.39 (0.10-1.09)† | 5.59 (0.20-20.94) | 2.05 (0.08-20.94) | 0.59 (0.02-30.61)* | 18.45 (0.97-20.94) | 6.59 (0.23-20.94) | 0.53 (0.24-2.32) | **0.009** |
| IgG 22F | 2.03 (0.19-19.00) | 0.71 (0.02-18.40) | 0.29 (0.01-1.89)† | 2.86 (0.19-19.00) | 0.71 (0.02-19.00) | 0.38 (0.01-28.65)* | 11.71 (0.28-19.00) | 2.62 (0.08-19.00) | 0.32 (0.08-41.62) | **0.041** |
| IgG 23F | 1.73 (0.12-11.90) | 0.53 (0.01-11.90) | 0.28 (0.01-1.00) | 2.21 (0.12-11.90) | 1.01 (0.01-11.90) | 0.38 (0.04-6.82) | 11.90 (0.48-11.90) | 2.46 (0.08-11.90) | 0.40 (0.09-1.00) | 0.228 |
| IgG 33F | 10.22 (0.21-21.32) | 4.26 (0.02-21.32) | 0.50 (0.01-4.70)† | 6.39 (0.21-21.32) | 2.64 (0.02-21.32) | 0.56 (0.04-18.66)† | 21.32 (0.72-21.32) | 11.59 (0.86-21.32) | 0.87 (0.29-4.92)* | **0.011** |

Significant *p*-values are in **bold**. *P*-values show differences in median fold changes between groups and are calculated by independent samples Kruskal-Wallis test. *is significantly higher than †. PLWH = people living with HIV.

## **Supplementary Table 6.** Changes in serotype-specific antibody concentrations between M4 and M36 in patients on immunosuppressive therapy.

|  | Biological immunomodulators | | | Conventional immunomodulators | | | Combination therapy | | | Switched | | |  |
| --- | --- | --- | --- | --- | --- | --- | --- | --- | --- | --- | --- | --- | --- |
| Serotype | **M4** | **M36** | **Median fold change** | **M4** | **M36** | **Median fold change** | **M4** | **M36** | **Median fold change** | **M4** | **M36** | **Median fold change** | ***p*-value** |
| IgG 1 | 1.59 (0.17-17.00) | 0.75 (0.02-17.00) | 0.39 (0.10-10.40) | 2.31 (0.17-17.00) | 1.11 (0.08-8.07) | 0.51 (0.14-5.55) | 1.78 (0.21-17.00) | 0.41 (0.03-17.00) | 0.40 (0.06-1.06) | 2.19 (0.22-5.31) | 0.47 (0.06-2.01) | 0.22 (0.10-1.32) | 0.095 |
| IgG 2 | 9.40 (0.48-49.26) | 5.27 (0.08-49.26) | 0.48 (0.03-12.00) | 8.41 (0.48-49.26) | 2.10 (0.21-49.26) | 0.54 (0.14-8.11) | 10.72 (0.48-49.26) | 4.25 (0.08-49.26) | 0.22 (0.10-1.32) | 29.94 (0.63-49.2)6 | 8.77 (0.18-47.40) | 0.28 (0.15-8.01) | 0.527 |
| IgG 3 | 0.55 (0.06-2.90) | 0.21 (0.02-2.29) | 0.39 (0.11-5.66) | 0.77 (0.05-2.90) | 0.25 (0.01-2.32) | 0.46 (0.13-0.96) | 0.32 (0.03-2.90) | 0.13 (0.01-2.90) | 0.39 (0.06-10.40) | 0.38 (0.17-2.04) | 0.21 (0.01-2.90) | 0.37 (0.05-4.17) | 0.472 |
| IgG 4 | 0.60 (0.09-6.66) | 0.22 (0.01-6.66) | 0.33 (0.03-9.94) | 1.29 (0.07-6.66) | 0.39 (0.01-6.66) | 0.43 (0.07-19.13) | 0.86 (0.07-6.66) | 0.22 (0.03-6.66) | 0.52 (0.05-17.22) | 1.44 (0.21-6.66) | 0.36 (0.03-6.66) | 0.32 (0.09-1.00) | 0.821 |
| IgG 5 | 1.17 (0.15-15.02) | 0.50 (0.03-15.02) | 0.36 (0.07-9.50) | 1.81 (0.15-15.02) | 0.62 (0.02-15.02) | 0.51 (0.051.98) | 1.46 (0.15-15.02) | 0.29 (0.03-15.02) | 0.39 (0.04-1.00) | 1.57 (0.15-15.02) | 0.48 (0.06-11.86) | 0.41 (0.12-1.02) | 0.361 |
| IgG 6A | 2.60 (0.08-7.86) | 1.45 (0.03-7.86) | 0.43 (0.10-13.46) | 2.06 (0.08-7.86) | 0.97 (0.02-7.86) | 0.49 (0.12-41.71) | 2.34 (0.08-7.86) | 1.03 (0.03-7.86) | 0.49 (0.06-1.55) | 3.47 (0.14-7.86) | 1.59 (0.02-7.86) | 0.59 (0.16-1.00) | 0.743 |
| IgG 6B | 2.70 (0.18-18.10) | 1.08 (0.03-18.10) | 0.36 (0.12-5.84) | 1.78 (0.18-18.10) | 0.85 (0.02-18.10) | 0.38 (0.10-3.49) | 2.42 (0.18-18.10) | 1.16 (0.04-18.10) | 0.40 (0.06-2.66) | 1.60 (0.18-18.10) | 1.17 (0.03-14.67) | 0.30 (0.16-0.85) | 0.633 |
| IgG 7 | 5.70 (0.22-16.60) | 2.42 (0.11-16.60) | 0.44 (0.06-10.97) | 4.36 (0.16-16.60) | 1.87 (0.04-10.46) | 0.55 (0.16-2.19) | 5.06 (0.33-16.60) | 1.26 (0.10-16.60) | 0.36 (0.04-1.17) | 5.92 (0.48-16.60) | 3.45 (0.20-10.19) | 0.39 (0.14-4.05) | 0.073 |
| IgG 8 | 5.41 (0.38-28.48) | 1.46 (0.05-28.48) | 0.49 (0.06-3.88) | 3.92 (0.28-28.48) | 1.21 (0.17-28.48) | 0.53 (0.22-1.14) | 7.18 (0.46-28.48) | 1.53 (0.08-21.41) | 0.48 (0.10-3.86) | 15.63(0.33-28.48) | 5.25 (0.13-28.48) | 0.39 (0.08-1.92) | 0.505 |
| IgG 9N | 3.57 (0.30-14.06) | 1.28 (0.07-14.06) | 0.35 (0.03-5.04) | 3.31 (0.18-14.06) | 1.41 (0.07-14.06) | 0.49 (0.09-2.84) | 3.88 (0.14-14.06) | 1.22 (0.08-14.06) | 0.44 (0.18-3.75) | 1.80 (0.17-14.06) | 0.87 (0.03-10.63) | 0.22 (0.06-1.13) | 0.253 |
| IgG 9V | 1.82 (0.13-12.88) | 0.74 (0.02-9.54) | 0.46 (0.05-6.16) | 1.54 (0.13-12.88) | 0.77 (0.04-12.88) | 0.59 (0.15-3.47) | 1.80 (0.13-12.88) | 0.83 (0.05-7.13) | 0.45 (0.14-2.04) | 1.67 (0.13-12.88) | 0.77 (0.06-11.02) | 0.44 (0.12-1.26) | 0.412 |
| IgG 10A | 2.34 (0.25-25.96) | 1.14 (0.05-25.96) | 0.37 (0.12-5.92)† | 4.31 (0.25-25.96) | 2.37 (0.03-25.96) | 0.58 (0.10-1.25)* | 2.35 (0.25-25.96) | 0.85 (0.03-25.96) | 0.44 (0.04-4.41) | 1.07 (0.25-25.96) | 0.22 (0.03-22.21) | 0.31 (0.00-0.90) | **0.017** |
| IgG 11A | 3.22 (0.17-10.16) | 0.85 (0.08-10.16) | 0.44 (0.06-5.47) | 3.21 (0.15-10.16) | 1.61 (0.16-10.16) | 0.61 (0.27-1.65) | 2.92 (0.10-10.16) | 1.10 (0.03-10.16) | 0.57 (0.07-98.00) | 3.45 (0.10-10.16) | 1.40 (0.06-10.16) | 0.54 (0.10-2.78) | 0.153 |
| IgG 12F | 0.61 (0.05-4.42) | 0.34 (0.06-4.42) | 0.90 (0.13-85.78) | 0.86 (0.04-4.42) | 0.43 (0.02-4.42) | 0.94 (0.07-5.66) | 0.39 (0.06-4.42) | 0.26 (0.03-4.42) | 0.67 (0.06-12.70) | 1.56 (0.05-4.42) | 0.33 (0.07-4.42) | 0.37 (0.02-5.65) | 0.293 |
| IgG 14 | 13.78 (0.74-75.98) | 4.36 (0.16-66.57) | 0.43 (0.10-32.27) | 9.55 (0.74-75.98) | 6.88 (0.07-75.98) | 0.66 (0.12-2.64) | 11.51 (0.74-75.98) | 3.50 (0.14-75.98) | 0.41 (0.05-13.01) | 15.63 (1.48-75.9)8 | 4.65 (0.35-52.93) | 0.33 (0.08-0.89) | 0.070 |
| IgG 15B | 7.76 (0.33-33.88) | 2.90 (0.09-33.88) | 0.40 (0.02-27.92) | 6.11 (0.33-33.88) | 3.78 (0.03-29.68) | 0.66 (0.19-12.66) | 4.38 (0.33-33.88) | 1.76 (0.07-32.06) | 0.43 (0.08-31.91) | 9.69 (0.56-33.88) | 5.05 (0.04-28.85) | 0.49 (0.00-6.34) | 0.536 |
| IgG 17F | 4.92 (0.17-17.02) | 1.39 (0.06-17.02) | 0.31 (0.09-7.97) | 2.60 (0.17-17.02) | 1.30 (0.03-17.02) | 0.53 (0.17-4.43) | 2.96 (0.17-17.02) | 0.89 (0.02-17.02) | 0.40 (0.04-102.40) | 10.74 (0.28-17.02) | 3.14 (0.14-17.02) | 0.43 (0.10-2.00) | 0.396 |
| IgG 18C | 5.39 (0.14-14.60) | 1.87 (0.10-14.60) | 0.50 (0.03-3.05) | 5.68 (0.14-14.60) | 2.91 ( 0.09-14.60) | 0.69 (0.15-2.79) | 6.11 (0.16-14.60) | 1.69 (0.15-14.60) | 0.54 (0.10-1.55) | 7.91 (0.14-14.60) | 3.51 (0.05-14.60) | 0.45 (0.14-1.84) | 0.273 |
| IgG 19A | 5.17 (0.27-27.74) | 2.67 (0.27-27.74) | 0.45 (0.08-3.45) | 5.65 (0.33-27.74) | 2.73 (0.20-27.74) | 0.52 (0.06-7.75) | 6.71 (0.49-27.74) | 2.40 (0.23-27.74) | 0.44 (0.05-1.87) | 19.22 (0.45-27.74) | 2.85 (0.10-24.32) | 0.32 (0.05-0.90) | 0.070 |
| IgG 19F | 2.80 (0.29-29.20) | 2.27 (0.08-27.85) | 0.40 (0.13-4.25) | 3.15 (0.38-29.22) | 1.73 (0.13-28.12) | 0.47 (0.15-2.53) | 6.82 (0.30-26.57) | 2.31 (0.12-14.39) | 0.38 (0.02-1.42) | 5.26 (0.82-29.22) | 1.32 (0.11-19.24) | 0.34 (0.04-0.90) | 0.152 |
| IgG 20 | 8.06 (0.24-20.94) | 2.62 (0.08-20.94) | 0.43 (0.04-5.45) | 3.80 (0.20-20.94) | 2.02 (0.19-20.94) | 0.71 (0.06-1.56) | 5.41 (0.35-20.94) | 1.74 (0.11-20.94) | 0.62 (0.02-30.61) | 3.62 (0.40-20.94) | 1.20 (0.14-20.94) | 0.67 (0.08-1.65) | 0.623 |
| IgG 22F | 3.06 (0.19-19.00) | 0.94 (0.02-11.18) | 0.34 (0.11-1.64) | 3.68 (0.19-19.00) | 0.97 (0.02-19.00) | 0.47 (0.10-2.08) | 1.83 (0.19-19.00) | 0.53(0.05-19.00) | 0.41 (0.02-28.65) | 2.84 (0.19-19.00) | 0.73 (0.03-10.16) | 0.36 (0.01-1.48) | 0.234 |
| IgG 23F | 2.05 (0.12-11.90) | 1.54 (0.01-11.90) | 0.39 (0.04-3.54) | 1.80 (0.12-11.90) | 0.79 (0.03-11.83) | 0.41 (0.13-6.82) | 2.09 (0.12-11.90) | 0.67 (0.02-6.44) | 0.32 (0.08-1.55) | 11.38 (1.15-11.90) | 3.57 (0.23-11.90) | 0.44 (0.07-1.10) | 0.112 |
| IgG 33F | 5.49 (0.21-21.32) | 2.80 (0.02-21.32) | 0.48 (0.10-18.66) | 7.98 (0.21-21.32) | 4.43 (0.06-21.32) | 0.64 (0.17-2.25) | 6.06 (0.26-21.32) | 1.49 (0.11-21.32) | 0.52 (0.04-13.31) | 6.82 (0.83-21.32) | 3.92 (0.62-21.32) | 0.64 (0.13-3.99) | 0.688 |

Significant *p*-values are in **bold**. *P*-values show differences in median fold changes between groups and are calculated by independent samples Kruskal-Wallis test. *is significantly higher than †.

Supplementary Table 7. Serologic response on M36 stratified by relevant variables among PLWH.

|  |  | **Serologic response at M36** | | | | |
| --- | --- | --- | --- | --- | --- | --- |
|  | **Total** | **Poor** | | **Moderate** | **Sufficient** | **Good** |
| **Age, *n* (%)** |  |  | |  |  |  |
| 18-49 years | 23 | 10/23 (44%) | | 13/23 (57%) | 6/23 (26%) | 4/23 (17%) |
| ≥ 50 years | 32 | 16/32 (50%) | | 16/32 (50%) | 3/32 (9%) | 1/32 (3%) |
| **Sex, *n* (%)** |  |  | |  |  |  |
| Male | 46 | 21/45 (46%) | | 25/46 (54%) | 7/46 (15%) | 5/46 (11%) |
| Female | 9 | 5/9 (56%) | | 4/9 (45%) | 2/9 (22%) | 0/9 (0%) |
| **Time since HIV-diagnosis, *n* (%)** |  |  | |  |  |  |
| < 10 months | 30 | 15/30 (50%) | | 15/30 (50%) | 4/30 (13%) | 2/30 (7%) |
| ≥ 10 months | 25 | 11/25 (44%) | | 14/25 (56%) | 5/25 (20%) | 3/25 (12%) |
| **AIDS at diagnosis, *n* (%)** |  |  | |  |  |  |
| No | 53 | 24/53 (45%) | | 29/53 (55%) | 9/53 (17%) | 5/53 (9%) |
| Yes | 2 | 2/2 (100%) | | 0/2 (0%) | 0/2 (0%) | 0/2 (0%) |
| **Viral load detectable at M0, *n* (%)** |  |  | |  |  |  |
| No | 49 | 20/49 (41%) | | 29/49 (59%) | 9/49 (18%) | 5/49 (10%) |
| Yes | 6 | 6/6 (100%) | | 0/6 (0%) | 0/6 (0%) | 0/6 (0%) |
| **CD4+ T cell number at M0, cells/mm^3^, *n* (%)** |  |  | |  |  |  |
| <400 | 13 | 6/13 (46%) | 7/13 (54%) | | 2/13 (15%) | 2/13 (15%) |
| ≥400 | 42 | 20/42 (48%) | 22/42 (52%) | | 7/42 (17%) | 3/42 (7%) |
| **Nadir CD4+ T cell number, cells/mm^3^, *n* (%)** |  |  |  | |  |  |
| <200 | 17 | 11/17 (65%) | 6/17 (35%) | | 1/17 (6%) | 0/17 (100%) |
| ≥200 | 29 | 11/29 (38%) | 18/29 (62%) | | 5/29 (17%) | 2/29 (7%) |
| **Serological response at M4, *n* (%)** |  |  |  | |  |  |
| <1.3 μg/mL for 17/24 serotypes | 28 | 21/28 (75%) | 7/28 (25%) | | 0/28 (0%) | 0/28 (0%) |
| ≥1.3 μg/mL for 17/24 serotypes | 22 | 3/22 (14%) | 19/22 (86%) | | 8/22 (36%) | 5/22 (23%) |
| **Pneumococcal booster vaccination, *n* (%)** |  |  |  | |  |  |
| No | 52 | 24/52 (46%) | 28/52 (54%) | | 9/52 (17%) | 5/52 (10%) |
| Yes | 3 | 2/3 (67%) | 1/3 (33%) | | 0/3 (0%) | 0/3 (0%) |

PCV13 = 13-valent pneumococcal conjugate vaccine (Prevenar 13®).

Supplementary Table 8. Factors associated with seroprotection at M36.

|  | Univariable regression | | Multivariable regression | |
| --- | --- | --- | --- | --- |
|  | OR (95% CI) | *p*-value | Adjusted OR (95% CI) | *p*-value |
| Age ≥ 50 years | 0.67 (0.24-1.86) | 0.445 | NE | NE |
| Male sex | 1.37 (0.54-3.46) | 0.510 | NE | NE |
| Inflammatory bowel disease | 1.99 (0.79-5.02) | 0.144 | NE | NE |
| Chronic kidney disease | 0.40 (0.09-1.82) | 0.234 | NE | NE |
| Combination therapy | 0.27 (0.08-0.98) | **0.046** | NE | NE |
| Biological immunomodulator | 1.27 (0.51-3.18) | 0.609 | NE | NE |
| Anti-CD20 therapy | 2.55 (0.22-29.9) | 0.454 | NE | NE |
| Switched | 3.27 (0.98-10.94) | 0.055 | 5.25 (1.23-22.51) | **0.026** |
| Seroprotected at M4 | 16.36 (2.08-129.05) | **0.008** | 18.40 (2.23-151.68) | **0.007** |
| Booster vaccine | 0.99 (0.11-8.92) | 0.993 | NE | NE |

Significant *p*-values are in **bold**. Predictors included in the multivariable regression model were selected based on prior literature and stepwise backward selection based on likelihood ratio and *p*-value <0.05. NE = not estimated. OR = odds ratio; NE = not estimated.

Supplementary Table 9. Serological responses at M36 among those with a good serological response at M36.

| Groups | Good serological response at M4 | Good serological response at M36 | Sufficient serological responses at M36 | Moderate serological response at M36 | Poor serological response at M36 |
| --- | --- | --- | --- | --- | --- |
| Overall | 54% (95/175) | 28% (27/95) | 46% (44/95) | 96% (91/95) | 42% (43/95) |
| PLWH | 44% (22/50) | 23% (5/22) | 36% (8/22) | 86% (19/22) | 14% (3/22) |
| Patients on bIM | 58% (19/33) | 21% (4/19) | 37% (7/19) | 95% (18/19) | 5% (1/19) |
| Patients on cIM | 154% (4/26) | 29% (4/14) | 57% (8/14) | 100% (14/14) | 0% (0/14) |
| Patients on combination therapy | 51% (18/35) | 11% (2/18) | 39% (7/18) | 100% (18/18) | 0% (0/18) |
| Switched group | 57% (8/14) | 63% (5/8) | 63% (5/8) | 100% (8/8) | 0% (0/8) |
| Controls | 82% (14/17) | 50% (7/14) | 64% (9/14) | 100% (14/14) | 0% (0/14) |

PLWH = people living with HIV; bIM = biological immunomodulator; cIM = conventional immunomodulator.

Supplementary Table 10. PCV13 and PPSV23-unique serotype-specific protection rates at DB7.

| Groups | Above cut-off for 9/13 (70%) of PCV13-serotypes | | | Above cut-off for 8/11 (70%) of PPSV23-unique serotypes | | |
| --- | --- | --- | --- | --- | --- | --- |
|  | **> 1.3 µg/mL** | **> 1.0 µg/mL** | **> 0.35 µg/mL** | **> 1.3 µg/mL** | **> 1.0 µg/mL** | **> 0.35 µg/mL** |
| PLWH | 6/10 (60%) | 7/10 (70%) | 9/10 (90%) | 5/10 (50%) | 7/10 (70%) | 10/10 (100%) |
| Patients on monotherapy | 5/14 (36%) | 6/14 (43%) | 13/14 (93%) | 8/14 (57%) | 9/14 (64%) | 14/14 (100%) |
| Patients on combination therapy | 5/9 (56%) | 5/9 (56%) | 9/9 (100%) | 5/9 (56%) | 6/9 (67%) | 9/9 (100%) |
| Controls | 2/3 (67%) | 2/3 (67%) | 3/3 (100%) | 2/3 (67%) | 3/3 (100%) | 3/3 (100%) |
| *p*-value | 0.570 | 0.460 | 0.735 | 0.868 | 0.469 | NA |

*P*-values show differences in proportions between groups and are calculated by the Chi-square test. PLWH = people living with HIV. NA = not applicable.

## **Supplementary Table 11.** Serotype-specific antibody concentrations at DB0 and DB7.

|  | Overall | | | PLWH | | Immunosuppressive therapy | | Controls | |
| --- | --- | --- | --- | --- | --- | --- | --- | --- | --- |
| Serotype | **DB0** | **DB7** | ***p*-value** | **DB0** | **DB7** | **DB0** | **DB7** | **DB0** | **DB7** |
| 1*† | 0.72 (0.27-3.03) | 2.02 (0.45-4.69) | 0.12 | 0.81 (0.14-7.57) | 6.06 (2.37-7.17) | 0.49 (0.21-4.45) | 1.11 (0.28-6.26) | 1.49 (0.55-2.23) | 2.09 (1.23-3.75) |
| 2 | 2.77 (1.39-12.15) | 3.21 (1.41-11.87) | 0.85 | 21.14 (13.77-49.26) | 17.7 (11.62-49.26) | 2.42 (1.39-10.01) | 3.4 (1.27-13.61) | 1.89 (0.91-4.51) | 2.18 (1.49-5.98) |
| 3*† | 0.13 (0.05-0.33) | 0.25 (0.09-0.52) | 0.06 | 0.17 (0.03-0.45) | 0.37 (0.03-0.96) | 0.12 (0.06-0.33) | 0.25 (0.09-0.49) | 0.13 (0.04-0.29) | 0.23 (0.16-0.59) |
| 4*† | 0.58 (0.11-1.82) | 1.14 (0.44-2.77) | **0.04** | 1.9 (0.64-4.73) | 6.66 (1-6.66) | 0.53 (0.1-1.65) | 0.95 (0.26-2.64) | 0.38 (0.11-1.42) | 1.2 (0.76-2.8) |
| 5*† | 0.52 (0.23-1.99) | 1.37 (0.41-3.49) | 0.09 | 14.48 (0.27-15.02) | 10.74 (2.77-15.02) | 0.43 (0.1-1.99) | 0.83 (0.26-3.37) | 0.58 (0.23-1.26) | 1.44 (0.69-2.21) |
| 6A*† | 1.19 (0.36-3.45) | 3.76 (1.19-6.34) | **0.01** | 0.53 (0.25-3.45) | 5.73 (0.32-7.86) | 1.4 (0.44-3.71) | 4.59 (0.71-6.62) | 1.19 (0.12-2.38) | 2.32 (1.57-6.14) |
| 6B*† | 1.59 (0.28-3.56) | 3.4 (1.6-5.82) | **0.02** | 0.16 (0.04-1.59) | 2.26 (0.05-3.66) | 2.06 (0.73-3.93) | 3.88 (1.61-7.62) | 0.44 (0.15-2.57) | 2.77 (1.3-4.14) |
| 7F*† | 1.58 (1.05-5.55) | 3.53 (1.86-6.12) | 0.06 | 3.4 (0.39-6.23) | 3.3 (0.86-8.62) | 1.58 (1.09-5.55) | 4.47 (1.82-6.33) | 1.52 (0.24-2.65) | 3.15 (1.91-3.77) |
| 8* | 1.39 (0.59-4.38) | 2.58 (0.86-6.15) | 0.31 | 5.33 (4.38-17.68) | 5.07 (4.26-16.28) | 0.81 (0.59-4.31) | 1.77 (0.85-6.12) | 2.31 (0.56-4.27) | 2.77 (0.77-8.01) |
| 9N | 1.21 (0.64-4.69) | 1.73 (0.66-5.15) | 0.65 | 1.21 (0.64-14.06) | 1.65 (0.61-14.06) | 1.04 (0.61-3.89) | 1.81 (0.4-4.88) | 1.28 (0.86-5.4) | 1.76 (1-6.44) |
| 9V*† | 0.7 (0.25-1.23) | 1.48 (0.49-3.21) | **0.01** | 0.6 (0.12-1.22) | 1.23 (1.19-1.53) | 0.7 (0.24-0.91) | 1.29 (0.36-2.9) | 1.12 (0.4-3.34) | 2.83 (1-4.29) |
| 10A* | 0.72 (0.37-2.8) | 2.09 (0.57-4.52) | 0.10 | 0.37 (0.19-4.02) | 0.74 (0.36-4.67) | 0.98 (0.44-4.28) | 2.37 (0.75-8.04) | 0.65 (0.4-1.68) | 1.6 (0.46-3.81) |
| 11A* | 1.96 (0.71-3.53) | 2.35 (1.13-4.6) | 0.11 | 0.91 (0.17-2.62) | 1.11 (0.21-2.35) | 2.08 (1-4) | 2.98 (1.72-6.55) | 1.27 (0.6-2.35) | 1.98 (0.97-3.44) |
| 12F* | 0.18 (0.04-1.2) | 0.25 (0.07-0.98) | 0.64 | 0.46 (0.01-4.42) | 1.55 (0.02-3.41) | 0.27 (0.04-1.37) | 0.36 (0.07-1) | 0.08 (0.06-0.34) | 0.08 (0.07-0.35) |
| 14*† | 2.88 (0.88-12.12) | 6.78 (1.82-17.22) | 0.14 | 8.3 (6.05-17.29) | 18.55 (7.44-20.17) | 1.7 (0.74-10.28) | 3 (0.79-14.99) | 2.88 (1.22-12.12) | 7.9 (5.02-16.88) |
| 15B* | 2.32 (0.8-6) | 3.6 (0.82-6.94) | 0.33 | 11.45 (5.57-18.27) | 13.06 (5.4-18.67) | 1.34 (0.8-5.05) | 1.91 (0.78-7.06) | 2.32 (0.5-5.83) | 2.87 (0.6-6.12) |
| 17F | 1.72 (0.73-6.42) | 1.85 (0.63-5.82) | 0.73 | 2.73 (1.15-16.09) | 2.31 (1.33-16.34) | 1.7 (0.56-4.14) | 1.77 (0.45-6.09) | 4.65 (0.91-7.02) | 2.87 (1.32-5.55) |
| 18C*† | 1.36 (0.7-3.28) | 2.93 (1.89-5.5) | **0.02** | 14.6 (1.15-14.6) | 9.93 (2.08-14.6) | 1.79 (0.87-2.86) | 2.55 (1.88-8.21) | 0.7 (0.33-1.88) | 3.01 (0.49-3.54) |
| 19A*† | 2.57 (1.06-7.35) | 3.92 (2.28-9.2) | 0.16 | 8.66 (5.4-19.9) | 8.51 (4.95-26.75) | 2.39 (1.06-4.22) | 3.15 (2.18-6.54) | 2.57 (0.96-8.5) | 3.88 (2.4-10.57) |
| 19F*† | 1.03 (0.43-2.97) | 2.65 (0.8-6.48) | **0.04** | 2.34 (0.74-3.93) | 3.19 (0.76-9) | 1.02 (0.42-1.89) | 2.54 (0.8-3.93) | 1.43 (0.46-7.12) | 4.61 (0.75-12.1) |
| 20 | 2.2 (0.6-4.25) | 2.04 (0.53-4.34) | 0.62 | 1.02 (0.18-5.99) | 1.04 (0.18-3.54) | 2.35 (0.91-6.91) | 2.53 (0.62-6.18) | 1.95 (0.53-3.39) | 1.75 (0.46-3.55) |
| 22F* | 0.72 (0.36-2.64) | 1.64 (0.51-3.16) | 0.13 | 1.77 (0.22-4.74) | 2.1 (0.21-8.71) | 0.63 (0.36-2) | 1.14 (0.43-3.27) | 1.05 (0.36-2.88) | 1.93 (0.78-2.97) |
| 23F*† | 0.49 (0.22-2.89) | 2.2 (0.57-3.41) | **0.01** | 0.73 (0.12-1.93) | 3.31 (2.13-11.9) | 0.45 (0.28-3.08) | 2.15 (0.51-3.41) | 0.66 (0.22-2.89) | 2.18 (0.41-3.42) |
| 33F* | 5.82 (1.86-11.09) | 5.84 (2.1-9.46) | 0.91 | 11.58 (11.09-19.28) | 11.7 (8.26-12.35) | 5.82 (2.03-10.08) | 5.78 (2.24-10.48) | 2.33 (1.32-8.39) | 4.01 (1.72-7.48) |

Significant *p*-values are in **bold**. * Serotype present in PCV20. † Serotypes present in PCV13.

## **Supplementary Table 12.** Rapid recall responses stratified by relevant variables.

|  |  | **Serologic response at DB7** | | | |
| --- | --- | --- | --- | --- | --- |
|  | **Total** | **Poor** | **Moderate** | **Sufficient** | **Good** |
| **Age, *n* (%)** |  |  |  |  |  |
| 18-49 years | 15 | 0/15 (0%) | 15/15 (100%) | 9/15 (60%) | 4/15 (27%) |
| ≥ 50 years | 21 | 1/21 (5%) | 20/21 (95%) | 9/21 (43%) | 6/21 (29%) |
| **Sex, *n* (%)** |  |  |  |  |  |
| Male | 24 | 1/24 (4%) | 23/24 (96%) | 13/24 (54%) | 8/24 (33%) |
| Female | 12 | 0/12 (0%) | 12/12 (100%) | 5/12 (42%) | 2/12 (17%) |
| **Group, *n* (%)** |  |  |  |  |  |
| PLWH | 10 | 1/10 (10%) | 9/10 (90%) | 7/10 (70%) | 4/10 (40%) |
| Immunosuppressive monotherapy | 14 | 0/14 (0%) | 14/14 (100%) | 4/14 (29%) | 2/14 (14%) |
| Immunosuppressive combination therapy | 9 | 0/9 (0%) | 9/9 (100%) | 5/9 (56%) | 2/9 (22%) |
| Controls | 3 | 0/3 (0%) | 3/3 (100%) | 2/3 (67%) | 2/3 (67%) |
| **Chronic kidney disease, *n* (%)** |  |  |  |  |  |
| No | 24 | 1/24 (4%) | 23/24 (96%) | 13/24 (54%) | 6/24 (25%) |
| Yes | 12 | 0/12 (0%) | 12/12 (100%) | 5/12 (42%) | 4/12 (33%) |
| **Time since HIV-diagnosis, *n* (%)** |  |  |  |  |  |
| < 10 months | 5 | 1/5 (20%) | 4/5 (80%) | 3/5 (60%) | 2/5 (40%) |
| ≥ 10 months | 5 | 0/5 (0%) | 5/5 (100%) | 4/5 (80%) | 2/5 (40%) |
| **CD4+ T cell number at M0, cells/mm^3^, *n* (%)** |  |  |  |  |  |
| <400 | 2 | 0/2 (0%) | 2/2 (100%) | 1/2 (50%) | 1/2 (50%) |
| ≥400 | 8 | 1/8 (13%) | 7/8 (88%) | 6/8 (75%) | 6/8 (38%) |
| **Nadir CD4+ T cell number, cells/mm^3^, *n* (%)** |  |  |  |  |  |
| <200 | 3 | 0/3 (0%) | 3/3 (100%) | 2/3 (67%) | 2/3 (67%) |
| ≥200 | 5 | 0/5 (0%) | 5/5 (100%) | 5/5 (100%) | 2/5 (40%) |
| **Underlying disease, *n* (%)** |  |  |  |  |  |
| IBD | 4 | 0/4 (0%) | 4/4 (100%) | 1/4 (25%) | 0/4 (0%) |
| Other | 19 | 0/19 (0%) | 19/19 (100%) | 8/19 (42%) | 4/19 (21%) |
| **Type of immunosuppressive therapy, *n* (%)** |  |  |  |  |  |
| Biological immunomodulators | 13 | 0/13 (0%) | 13/13 (100%) | 5/13 (39%) | 3/13 (23%) |
| Conventional immunomodulators | 10 | 0/10 (0%) | 10/10 (100%) | 4/10 (40%) | 1/10 (10%) |

None of the PLWH had AIDS at diagnosis and none had a detectable viral load. None of the patients on immunosuppressive therapy started immunosuppressive therapy after PCV13 (switched group). PLWH = people living with HIV; IBD = inflammatory bowel disease.

Supplementary Figure 1. Study procedures.


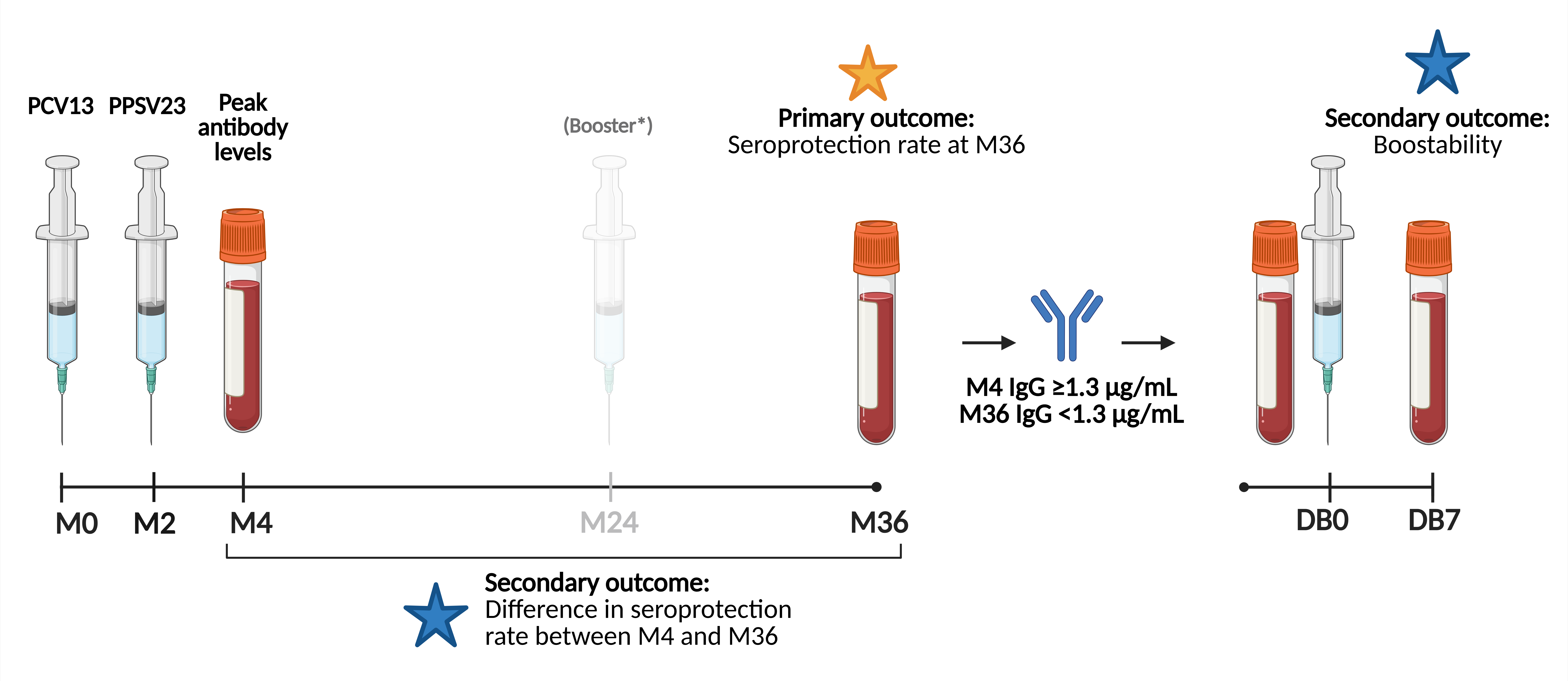
PCV13 = 13-valent pneumococcal conjugate vaccine (Prevenar 13®); PPSV23 = 23-valent polysaccharide vaccine (Pneumovax 23®). M depicts the months after PCV13. *Three people living with HIV (PLWH) received PCV13 + PPSV23 and two PLWH received PPSV23 only; Six patients on immunosuppressive therapy received PCV13 + PPSV23. Created in Biorender.

Supplementary Figure 2. Flowchart of study participants.


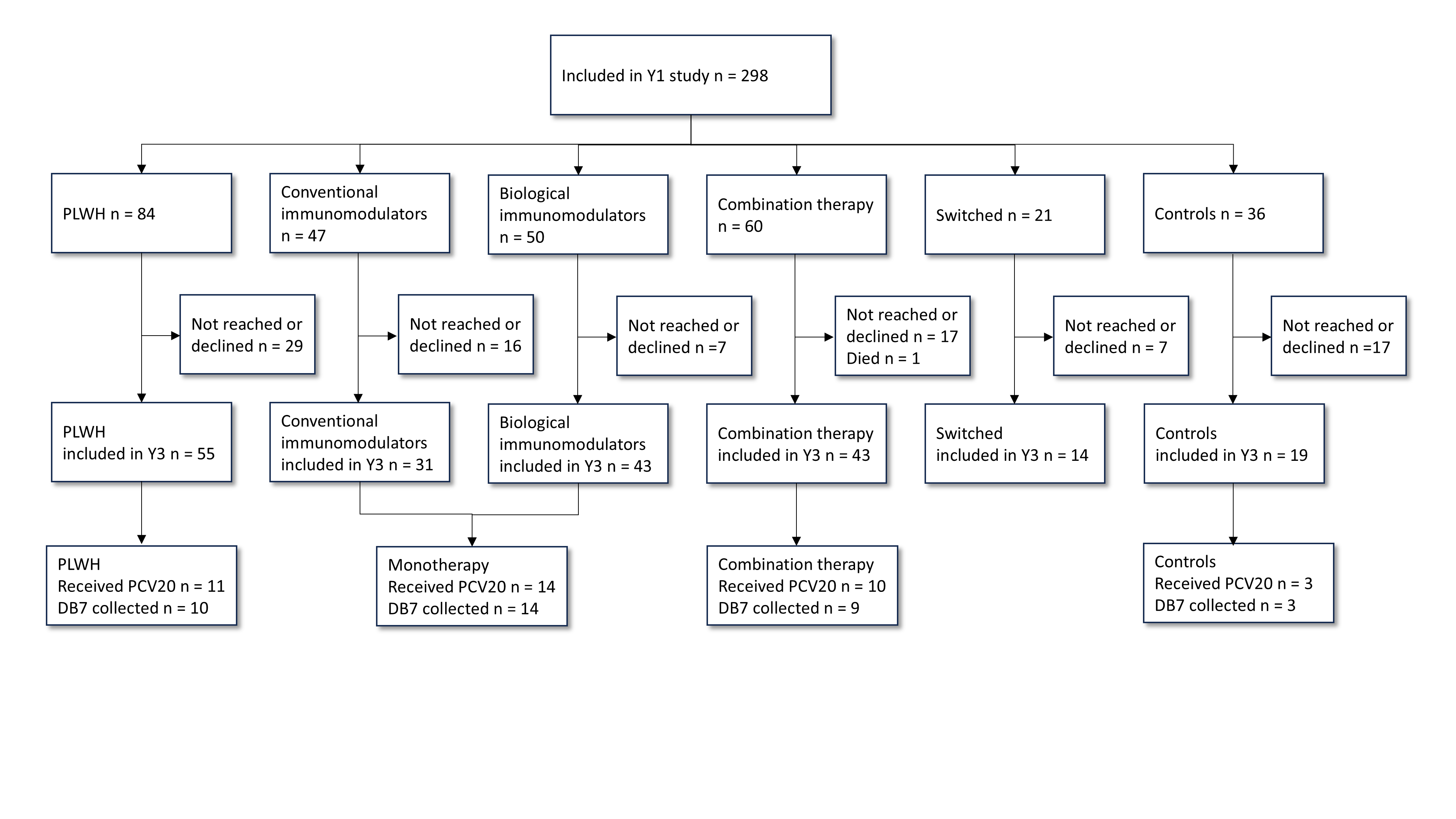


Y1 = year one; Y3 = year three; PLWH = people living with HIV. PCV20 = 20-valent pneumococcal conjugate vaccine (Prevenar 20®); DB0 = seven days after PCV20.
